# A bibliometric analysis of COVID-19 publications in neurology by using the visual mapping method

**DOI:** 10.3389/fpubh.2022.937008

**Published:** 2022-07-26

**Authors:** Qian Zhang, Jian Li, Ling Weng

**Affiliations:** ^1^Department of Neurosurgery, Xiangya Hospital, Central South University, Changsha, China; ^2^Hypothalamic-Pituitary Research Center, Xiangya Hospital, Central South University, Changsha, China; ^3^National Clinical Research Center for Geriatric Disorders, Xiangya Hospital, Central South University, Changsha, China; ^4^Hydrocephalus Center, Department of Neurosurgery, Xiangya Hospital, Central South University, Changsha, China; ^5^Department of Neurology, Xiangya Hospital, Central South University, Changsha, China

**Keywords:** COVID-19, neurology, bibliometric analysis, Citespace, VOSviewer

## Abstract

**Background:**

The characteristic symptom of coronavirus disease 2019 (COVID-19) is respiratory distress, but neurological symptoms are the most frequent extra-pulmonary symptoms. This study aims to explore the current status and hot topics of neurology-related research on COVID-19 using bibliometric analysis.

**Methods:**

Publications regarding neurology and COVID-19 were retrieved from the Web of Science Core Collection (WoSCC) on March 28 2022. The Advanced search was conducted using “TS = (‘COVID 19’ or ‘Novel Coronavirus 2019’ or ‘Coronavirus disease 2019’ or ‘2019-nCOV’ or ‘SARS-CoV-2’ or ‘coronavirus-2’) and TS = (‘neurology’or ‘neurological’ or ‘nervous system’ or ‘neurodegenerative disease’ or ‘brain’ or ‘cerebra’ or ‘nerve’)”. Microsoft Excel 2010 and VOSviewer were used to characterize the largest contributors, including the authors, journals, institutions, and countries. The hot topics and knowledge network were analyzed by CiteSpace and VOSviewer.

**Results:**

A total of 5,329 publications between 2020 and 2022 were retrieved. The United States, Italy, and the United Kingdom were three key contributors to this field. Harvard Medical School, the Tehran University of Medical Sciences, and the UCL Queen Square Institute of Neurology were the major institutions with the largest publications. Josef Finsterer from the University of São Paulo (Austria) was the most prolific author. Tom Solomon from the University of Liverpool (UK) was the most cited author. *Neurological Sciences* and *Frontiers in Neurology* were the first two most productive journals, while *Journal of Neurology* held the first in terms of total citations and citations per publication. Cerebrovascular diseases, neurodegenerative diseases, encephalitis and encephalopathy, neuroimmune complications, neurological presentation in children, long COVID and mental health, and telemedicine were the central topics regarding the neurology-related research on COVID-19.

**Conclusion:**

Neurology-related research on COVID-19 has attracted considerable attention worldwide. Research topics shifted from “morality, autopsy, and telemedicine” in 2020 to various COVID-19-related neurological symptoms in 2021, such as “stroke,” “Alzheimer's disease,” “Parkinson's disease,” “Guillain–Barre syndrome,” “multiple sclerosis,” “seizures in children,” and “long COVID.” “Applications of telemedicine in neurology during COVID-19 pandemic,” “COVID-19-related neurological complications and mechanism,” and “long COVID” require further study.

## Introduction

The coronavirus disease (COVID-19) pandemic continues, with new cases continuing to rise globally (1). As of 24 April 2022, more than 500 million confirmed cases and more than 6 million deaths have been recorded (https://covid19.who.int/). Although the characteristic symptom of COVID-19 is respiratory distress, neurologic symptoms are the most common extra-pulmonary symptoms (2), such as headaches, anosmia, cognitive dysfunction, and acute cerebrovascular disorders, which have been reported in numerous studies (3, 4). These symptoms appear to be a combination of nonspecific complications of systemic disease, effects of direct viral infection, or inflammation of the neurological and vascular systems (3). Varatharaj et al. conducted a national inter-professional surveillance study, including 153 patients with acute neurological and psychiatric complications associated with COVID-19. Of these, 62% (77/125) of them presented with cerebrovascular events, including 57 with ischemic strokes, nine with cerebral hemorrhages, and one with central nervous system vacuity. Overall, 31% (39/125) of the patients presented with altered mental status, including nine with unspecified encephalopathy and seven with encephalitis (5). In addition, the long-term neurological symptoms of acute sequelae of COVID-19 or “long COVID” can affect the entire spectrum of COVID-19 patients, ranging from mild to severe. Similar to acute COVID-19, long COVID may involve multiple organs and affect many systems, especially the neurological system. Symptoms of long COVID include fatigue, dyspnea, cardiac abnormalities, cognitive impairment, sleep disturbances, PTSD symptoms, muscle pain, distraction, and headaches (6). Considering the variety of neurological symptoms and complications of COVID-19, scholars have conducted many reviews on the association between COVID-19 and neurology (3, 7, 8). However, there are still some shortcomings that need to be addressed: (1) Most reviews use a meta-based approach, and this type of review does not provide an overview of all neurological research publications related to COVID-19. (2) Samples in some systematic reviews are subjectively screened, with small sample sizes. (3) The studies included were not comprehensive (e.g., reviews of randomized controlled trials or those with focus only on specific and limited aspects).

Bibliometric analysis is a widely accepted quantitative technique for analyzing big data of articles in a given field (9). It typically applies bibliometric tools (e.g., Bibliometrix R, Gephi, Pajek, CiteSpace, and VOSviewer) to analyze publication trends, popular articles, major contributors, central themes, and frontier topics in a given field (10). Several bibliometric analyses exist on different disciplines of COVID-19, such as pediatrics (11), urology (12), and rheumatology (13). These works provide readers and researchers with an overview of COVID-19-related research in specific disciplines. However, no study provides a state-of-the-art overview of neurology-related research on COVID-19. Therefore, in this study, we analyzed the neurology-related research on COVID-19 based on the WoSCC using the most popular bibliometric tools CiteSpace and VOSviewer (14). The aim of this article is to answer the following research questions.

- Question 1: What are the published trends in neurology-related research on COVID-19?
- Question 2: What are the most influential articles and major contributing authors, institutions, countries, and journals for neurology-related research on COVID-19?
- Question 3: Who are the potential collaborators (authors, institutions, countries/regions) for neurology-related research on COVID-19?
- Question 4: What are the important topics and frontier themes in neurology-related research on COVID-19?

## Materials and Methods

### Search strategy

Just as other bibliometric analysis steps, we began our search with the keyword “Neurology” or in the category of “Neuroscience and Neurology” on the WoSCC. However, the results did not include all publications on neurology and the COVID-19 discipline. Therefore, we used the following keywords: TS=(“COVID 19” or “Novel Coronavirus 2019” or “Coronavirus disease 2019” or “2019-nCOV” or “SARS-CoV-2” or “coronavirus-2”) and TS= (“neurology” or “neurology” or “neurological” or “neurodegenerative disease” or “brain” or “cerebral” or “neurological”) to filter publications in the field of neurology focused on COVID-19 or SARS-CoV-2. The online search was conducted on 28 March 2022 on the WoSCC. For this review, two researchers (Qian Zhang and Jian Li) independently searched the database, screening titles and abstracts and eliminating irrelevant articles. Any disagreements were resolved by discussing with the senior neurologist (Ling Weng) until consensus was reached.

### Data extraction and bibliometric analysis

We exported full records and cited references of all publications from the WoSCC. In addition, the bibliometric parameters (e.g., title, keywords, journal, publication year, citation, author, institution, country, and reference) were extracted. These data were then imported into Microsoft Excel 2010 (Redmond, WA, US) and VOSviewer (Leiden University, Leiden, and The Netherlands) to identify the most prolific contributors (e.g., prolific authors, institutions, and countries). In VOSviewer, node size is positively correlated with the number of articles. Co-authorship analysis was used to assess collaboration among different authors, countries, and institutions (15). The total link strength (TLS; the sum of link weights connected to a node) indicates the power of cooperation between two nodes. The width of links between two nodes is positively correlated with the cooperation strength. CiteSpace (version 5.8.R1) and VOSviewer were used to visualize keyword co-occurrence analysis and reference analysis (16).

## Results

### General data

**Figure 1:**
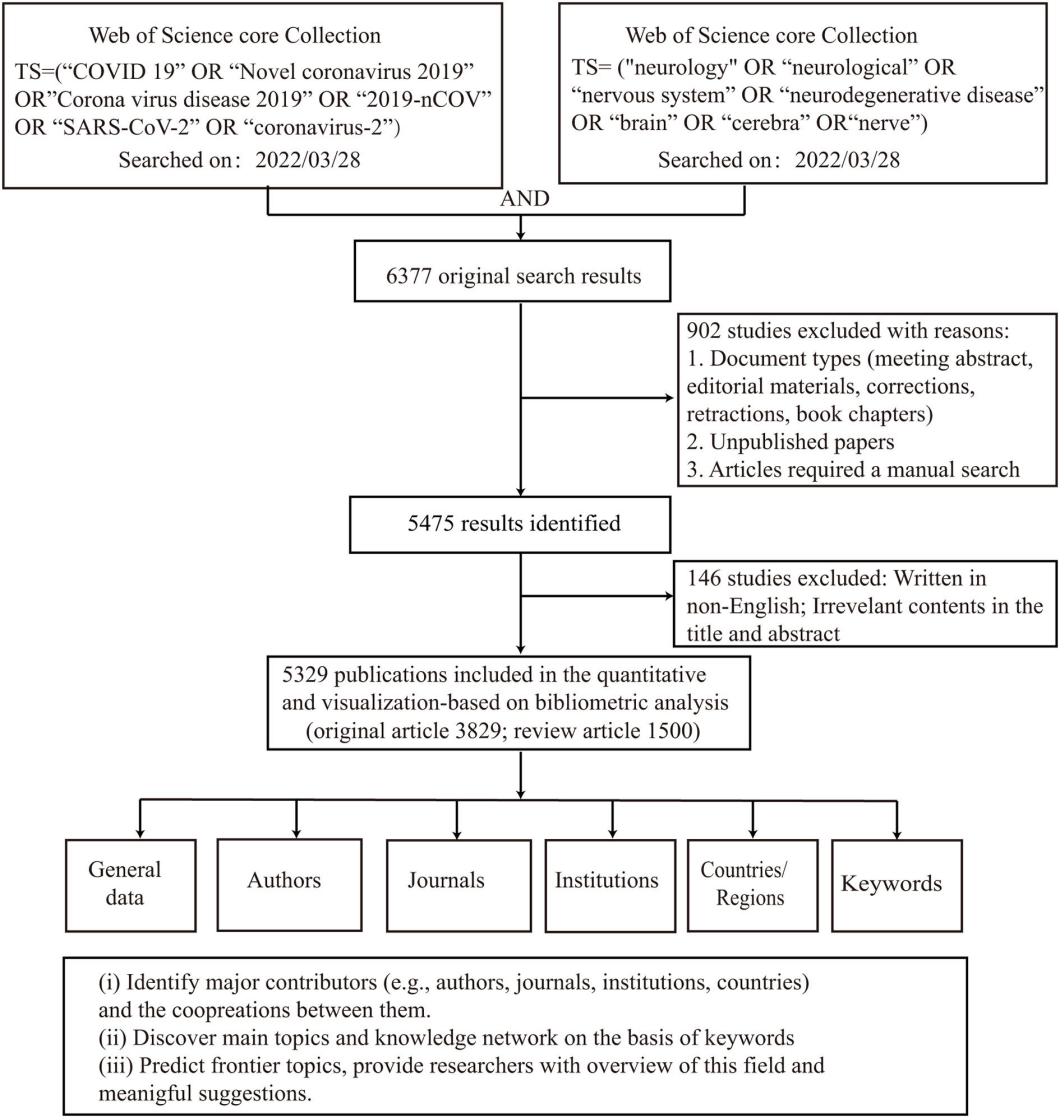
Flowchart of data screening and bibliometric analysis.

**Figure 2:**
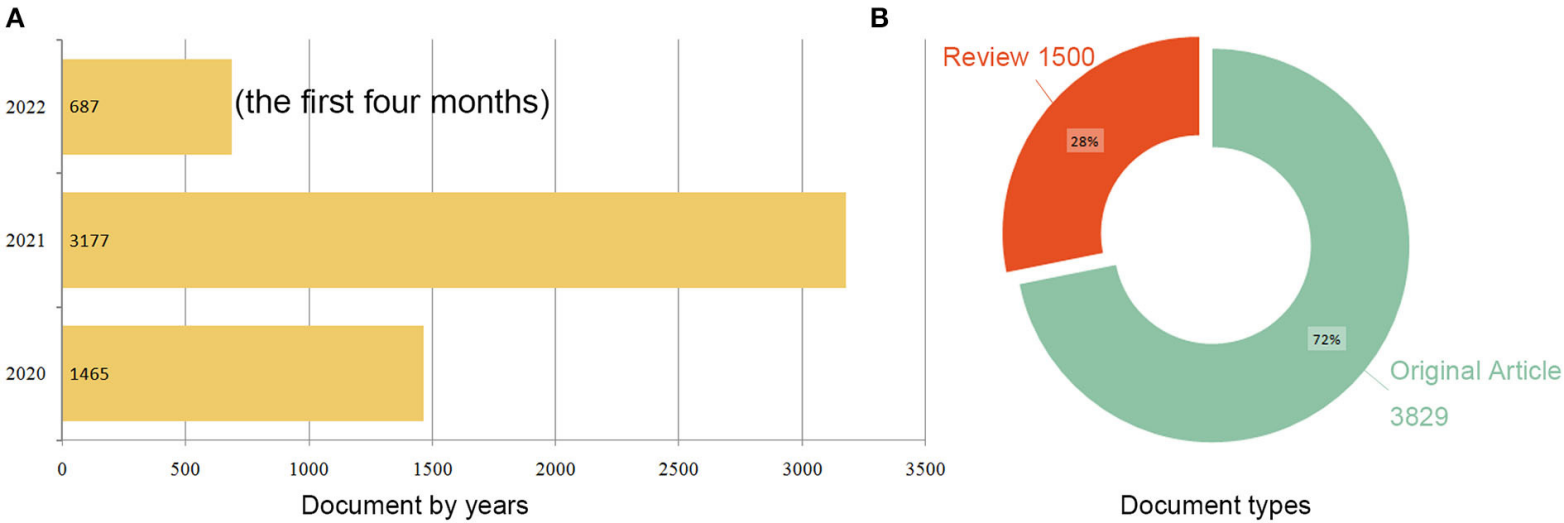
Distribution of publications by year **(A)** and type **(B)**.

Figure 1 illustrates the process of data screening and bibliometric analysis. After excluding articles written in languages other than English and limiting them to original articles and reviews, 5,341 publications remained. Of these, 12 articles containing the term “brain natriuretic peptide” and focusing on cardiovascular issues in patients with COVID-19 were excluded. Therefore, 5,329 publications were selected for further analysis, of which 72% were original articles (*n* = 3,829) and the rest were reviews (*n* = 1,500; Figure 2B). A total of 69,908 citations were received, with 13.12 citations per article and an H-index of 98. A total of 142 countries/regions, 7,684 institutions, 30,547 authors, and 1,646 journals contributed to these publications. Fudan University published the first article titled “A multicentre observational study on neonates exposed to SARS-CoV-2 in China: the Neo-SARS-CoV-2 study protocol” in China in March 2020 (17). The number of publications increased from 1,465 in 2020 to 3,177 in 2021 and 687 in the first 4 months of 2022 (Figure 2A).

### Top contributing countries

**Figure 3:**
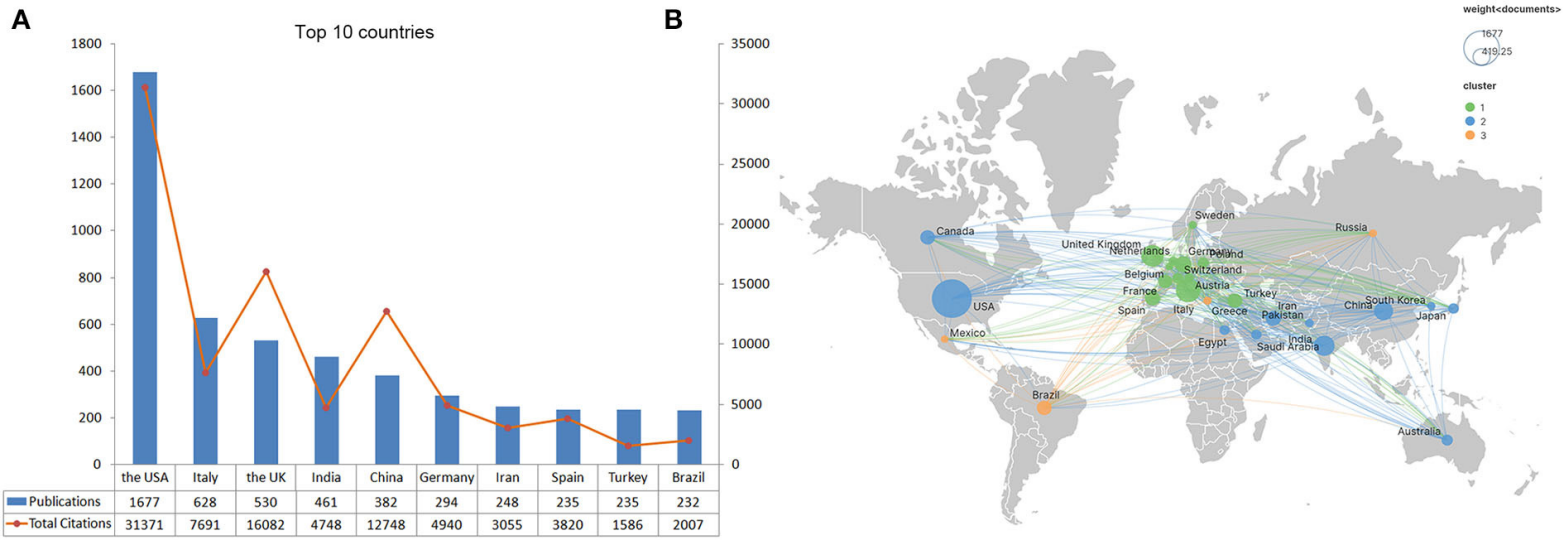
Top 10 prolific countries/regions and inter-national collaboration network of neurology-related research on COVID-19. **(A)** Number of publications and total citations for each country. **(B)** Collaboration among countries. Node size indicates the number of articles produced. The width of links indicated the cooperation strength.

The top 10 contributing countries are shown in Figure 3. The United States dominates the field, with 1,677 publications (31.4% of the total) and 31,371 total citations (44.8% of the total). Italy ranked second, with 628 publications and 7,691 total citations, and the United Kingdom ranked third, with 530 publications and 16,082 total citations (Figure 3A). We used VOSviewer to depict the international collaboration in this field. The minimum number of publications was set at 50. Finally, 28 countries were selected for visualization. The United States, Italy, the United Kingdom, India, and China are the largest nodes and more broadly connected, indicating their close collaboration and significant academic influence in the field (Figure 3B). The United Kingdom (115 TLS) and Canada (102 TLS) cooperate closely with the United States.

### Top contributing institutions

**Figure 4:**
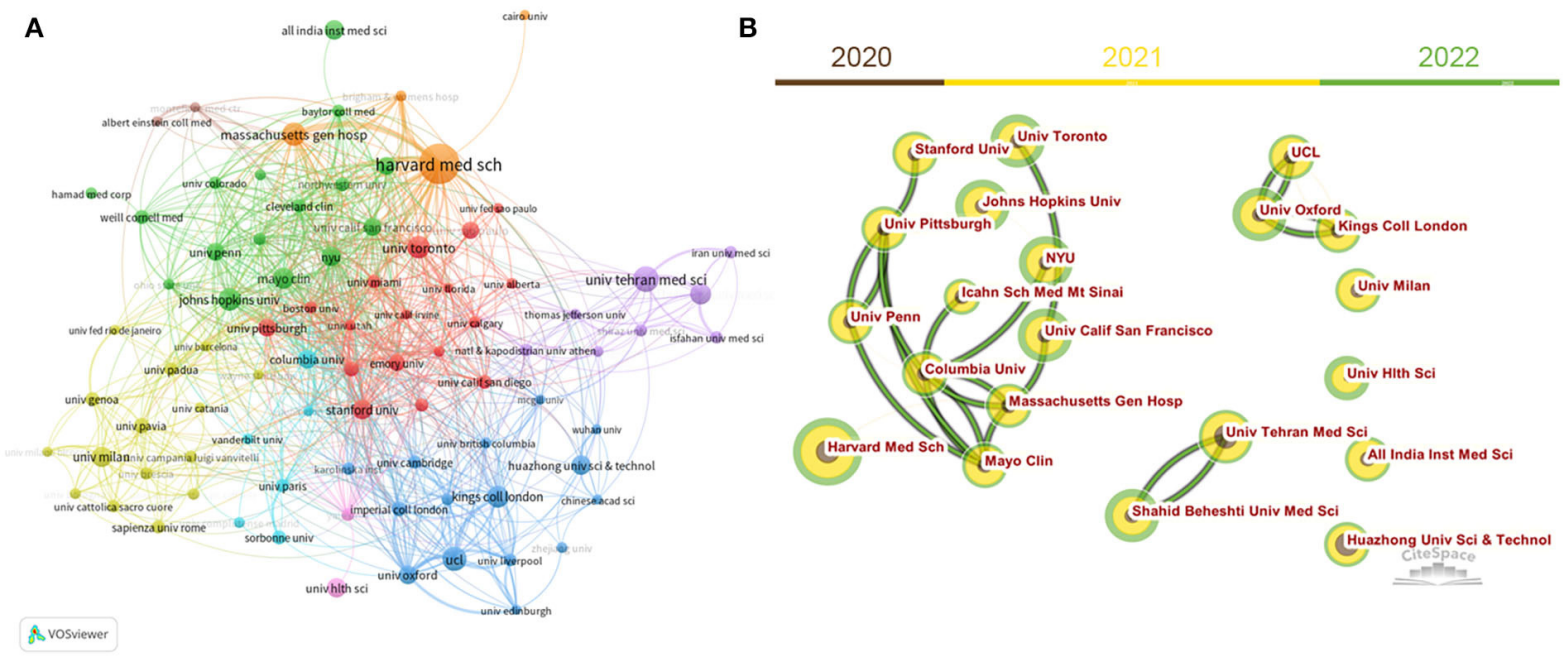
Inter-institution cooperative network built by VOSviewer **(A)** and CiteSpace **(B)**. Node size indicates the number of articles produced. The width of links indicates the cooperation strength.

Table 1 lists the top 10 most prolific and highly cited institutions for neurology-related research on COVID-19. Together, these institutions published 11.8% of the articles in the field. Specifically, Harvard Medical School (USA) ranked first, with 118 articles. The Tehran University of Medical Sciences (Iran) ranked second, with 68 articles. The UCL Queen Square Institute of Neurology ranked third, with 63 articles. Among the most influential institutions, the Huazhong University of Science and Technology (China), the UCL Queen Square Institute of Neurology (UK), and King's College London (UK) ranked the top three, with 6,162, 5,309, and 3,866 total citations, respectively. Figure 4A displays the network of collaborations among institutions. Harvard Medical School (US), Massachusetts General Hospital (US), and Mayo Clinic (US) are the central nodes in North America. King's College London (UK), the UCL Queen Square Institute of Neurology (UK), and the University of Liverpool (UK) present the central in the United Kingdom. The University of Milan plays an important role in Italy. The Tehran University of Medical Sciences (Iran) is a central node in the Middle East. Collaborations are more common among institutions that are geographically close to each other. As shown in Figure 4B, more collaborations occur among North American institutions.

**Table 1:** The top 10 most prolific institutions and highly cited institutions for neurological COVID-19.

**Institution**	**Publications**	**Total citations**	**Average citations**	**Country**
**Panel A: Institution with most publications**				
Harvard Med Sch	118	1,364	11.6	USA
Univ Tehran Med Sci	68	820	12.1	Iran
UCL, Queen Sq Inst Neurol	63	5,309	84.3	England
Massachusetts Gen Hosp	59	649	11.0	USA
Univ Toronto	58	757	13.1	Canada
Johns Hopkins Univ	56	660	11.8	USA
Kings Coll London	55	3,866	70.3	England
Mayo Clin	53	493	9.3	USA
Shahid Beheshti Univ Med Sci	53	678	12.8	Iran
Univ Milan	50	327	6.5	Italy
**Panel B: Institution with most citations**				
Huazhong Univ Sci & Technol	46	6,162	134.0	China
UCL, Queen Sq Inst Neurol	63	5,309	84.3	England
Kings Coll London	55	3,866	70.3	England
Univ Oxford	44	3,849	87.5	England
Univ Cambridge	29	2,855	98.4	England
Emory Univ	37	2,519	68.1	USA
Karolinska Inst	27	2,407	89.1	Sweden
Univ Calif Irvine	20	2,384	119.2	USA
Univ Liverpool	28	1,902	67.9	England
Harvard Med Sch	118	1,364	11.6	USA

### Top contributing authors

**Figure 5:**
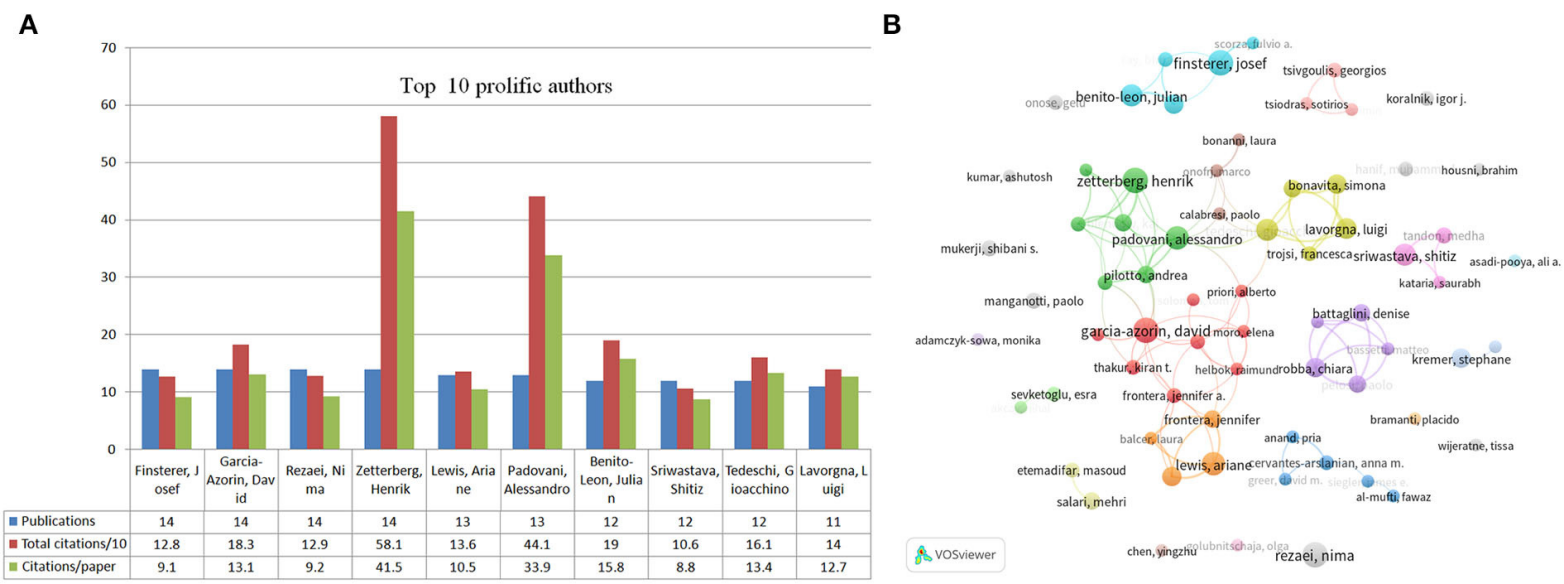
Top 10 most productive authors and collaboration among these authors. **(A)** Number of publications, total citations, and citations per publication in the top 10 prolific authors. **(B)** Collaboration among these prolific authors built by VOSviewer. Node size indicates the number of articles. The width of links indicates the cooperation strength.

Table 2 lists the top 10 contributing authors for neurology-related research on COVID-19. The most prolific authors (*n* = 14 publications) were Josef Finsterer from the University of São Paulo (Austria), David García-Azorín from Hospital Clinical University Valladolid (Spain), Nima Rezaei from the Tehran University of Medical Sciences (Iran), and Henrik Zetterberg from the Queen Square Institute of Neurology (UK). The most influential author, however, was Tom Solomon from the University of Liverpool (UK), with 1,100 total citations and 185 citations per article, followed by Henrik Zetterberg from Sahlgrenska University (UK), with 581 total citations and 41.5 citations per article, and Ali Asadi-Pooya from the Shiraz University of Medical Science (Iran), with 482 total citations and 80.3 citations per article. Co-authorship of authors in VOSviewer was used to identify collaborations among researchers. The minimum number of publications was 6. Ultimately, 69 authors were included in the visual analysis. Figure 5A shows top 10 prolific authors in this field. Figure 5B displays the author collaborative network, with Alessandro Padovani (Italy) being the first-tier author with 47 TLS. Gioacchino Tedeschi (Italy) and Andrea Pilotto (Italy) were the first to collaborate with 46 TLS and 43 TLS, respectively.

**Table 2:** The top 10 most prolific authors and highly cited authors for neurological COVID-19.

**Author**	**Publications**	**Total citations**	**Average citations**	**Latest reported institution**	**Country**
**Panel A:Authors with most publications**					
Finsterer, Josef	14	128	9.1	Univ Sao Paolo	Austria
Garcia-Azorin, David	14	183	13.1	Hosp Clin Univ Valladolid	Spain
Rezaei, Nima	14	129	9.2	Tehran University of Medical Sciences	Iran
Zetterberg, Henrik	14	581	41.5	UCL, Queen Sq Inst Neurol	England
Lewis, Ariane	13	136	10.5	New York University	USA
Padovani, Alessando	13	441	33.9	University of Brescia	Italy
Benito-Leon, Julian	12	190	15.8	Univ Hosp Octubre	Spain
Sriwastava, Shitiz	12	106	8.8	West Virginia University	USA
Tedeschi, Gioacchino	12	161	13.4	Universita della Campania Vanvitelli	Italy
Lavorgna, Luigi	11	140	12.7	Universita della Campania Vanvitelli	Italy
**Panel B:Authors with most citations**					
Solomon, Tom	6	1,110	185	University of Liverpool	England
Zetterberg, Henrik	14	581	41.5	Sahlgrenska University	Sweden
Asadi-Pooya, Ali A.	6	482	80.3	Shiraz University of Medical Science	Iran
Padovani, Alessandro	13	441	33.9	University of Brescia	Italy
Koralnik, Igor J.	7	441	63	Northwestern University	USA
Blennow, Kaj	9	391	43.4	University of Gothenburg	England
Kremer, Stephane	10	376	37.6	Hop Univ Strasbourg	France
Ashton, Nicholas J.	7	359	51.3	University of Gothenburg	England
Leonardi, Matilde	7	229	32.7	University of Gothenburg	England
Lersy, Francois	6	227	37.8	Univ Hosp Strasboug	France

### Top contributing journals

The top 10 active journals contributed 14.9% of the articles in this field. Table 3 lists the top 10 active journals in a descending numerical order. Specifically, the top three prolific journals are *Neurological Sciences* (*n* = 121), *Frontiers in Neurology* (*n* = 118), and *Cureus* (*n* = 109). However, in terms of impact, *Journal of Neurology* ranked first (with 1,535 total citations), followed by *Journal of Neuroscience* (*n* = 1,359) and *Frontiers in Neurology* (*n* = 1,217).

**Table 3:** Ranking of top 10 prolific journals for neurological COVID-19.

**Rank**	**Journal**	**Publications**	**Total citations**	**Average citations**	**Impact factor**	**Quartile in category**
1	Neurological Sciences	121	1,359	11.2	3.3073	Q2
2	Frontiers in Neurology	118	1,217	10.3	4.003	Q2
3	Cureus	109	378	3.5	NA	NA
4	BMJ Case Reports	92	614	6.7	NA	NA
5	Cureus Journal of Medical Science	88	981	11.1	NA	NA
6	Journal of Neurology	65	1,535	23.6	4.849	Q2
7	Journal of Clinical Medicine	58	347	6.0	4.24	Q1
8	European Journal of Neurology	55	974	17.7	6.089	Q1
9	Frontiers in Immunology	48	348	7.3	7.561	Q1
10	Journal of Neurovirology	45	520	11.6	2.643	Q3

### Highly cited articles

Almost half of the top 20 most cited publications are the review type (Table 4). Mao et al. produced the most cited article published in *JAMA Neurology* entitled “Neurologic Manifestations of Hospitalized Patients with Coronavirus Disease 2019 in Wuhan, China”, with 1,219 total citations (4). Most of the articles discussed neurological manifestations and complications related to COVID-19, such as stroke (18), meningitis/encephalitis (19), multiple sclerosis (20), and demyelinating diseases (21). Several publications discussed COVID-19-related mental health and neuropsychiatric manifestations (5, 22). Totally, five articles focused on the neuroinvasive mechanisms of SARS-CoV-2 (23–25).

**Table 4:** The top 20 cited articles on neurological COVID-19.

**Rank**	**Authors**	**Title**	**Citations**	**Journal**	**Publication**	**Type**
1	Mao, Ling	Neurologic Manifestations of Hospitalized Patients With Coronavirus Disease 2019 in Wuhan, China	4,511	JAMA Neurol.	2020	Article
2	Holmes, Emily A.	Multidisciplinary research priorities for the COVID-19 pandemic: a call for action for mental health science	2,219	Lancet Psychiatry	2020	Article
3	Li, Yan-Chao	The neuroinvasive potential of SARS-CoV2 may play a role in the respiratory failure of COVID-19 patients	1,316	J. Med. Virol.	2020	Review
4	Moriguchi, TK.	A first case of meningitis/encephalitis associated with SARS-Coronavirus-2	1,067	Int. J. Infect. Dis.	2020	Article
5	Gheblawi, Mahmoud	Angiotensin-Converting Enzyme 2: SARS-CoV-2 Receptor and Regulator of the Renin-Angiotensin System Celebrating the 20th Anniversary of the Discovery of ACE2	883	Circ.Res.	2020	Review
6	Ellul, Mark A.	Neurological associations of COVID-19	869	Lancet Neurol.	2020	Review
7	Wu, Yeshun	Nervous system involvement after infection with COVID-19 and other coronaviruses	861	Brain Behav. Immun.	2020	Review
8	Li, Meng-Yuan	Expression of the SARS-CoV-2 cell receptor gene ACE2 in a wide variety of human tissues	668	Infect. Dis. Poverty	2020	Article
9	Gonzalez-Sanguino, Clara	Mental health consequences during the initial stage of the 2020 Coronavirus pandemic (COVID-19) in Spain	558	Brain Behav. Immun.	2020	Article
10	Paterson, Ross W	The emerging spectrum of COVID-19 neurology: clinical, radiological and laboratory findings	456	Brain	2020	Article
11	Vivanti, Alexandre J.	Transplacental transmission of SARS-CoV-2 infection	467	Nat. Commun.	2020	Article
12	Zubair, Adeel S.	Neuropathogenesis and Neurologic Manifestations of the Coronaviruses in the Age of Coronavirus Disease 2019 A Review	402	JAMA Neurol.	2020	Review
13	Troyer, Emily A.	Are we facing a crashing wave of neuropsychiatric sequelae of COVID-19? Neuropsychiatric symptoms and potential immunologic mechanisms	393	Brain Behav. Immun.	2020	Review
14	Asadi-Pooya, Ali A.	Central nervous system manifestations of COVID-19: A systematic review	365	J. Neurol. Sci.	2020	Review
15	Matschke, Jakob	Neuropathology of patients with COVID-19 in Germany: a post-mortem case series	365	Lancet Neurol.	2020	Article
16	Meinhardt, JennyLorenz	Olfactory transmucosal SARS-CoV-2 invasion as a port of central nervous system entry in individuals with COVID-19	355	Nat. Neurosci.	2021	Article
17	Filatov, Asia	Neurological Complications of Coronavirus Disease (COVID-19): Encephalopathy	335	Cureus J Med Sci	2020	Article
18	Taquet, Maxime	6-month neurological and psychiatric outcomes in 236 379 survivors of COVID-19: a retrospective cohort study using electronic health records	330	Lancet Psychiatry	2021	Article
19	Avula, Akshay	COVID-19 presenting as stroke	329	Brain Behav. Immun.	2020	Article
20	Montalvan, V.	Neurological manifestations of COVID-19 and other coronavirus infections: A systematic review	256	Clin. Neurol. Neurosurg.	2020	Review

### Analysis of keywords and co-cited references

**Figure 6:**
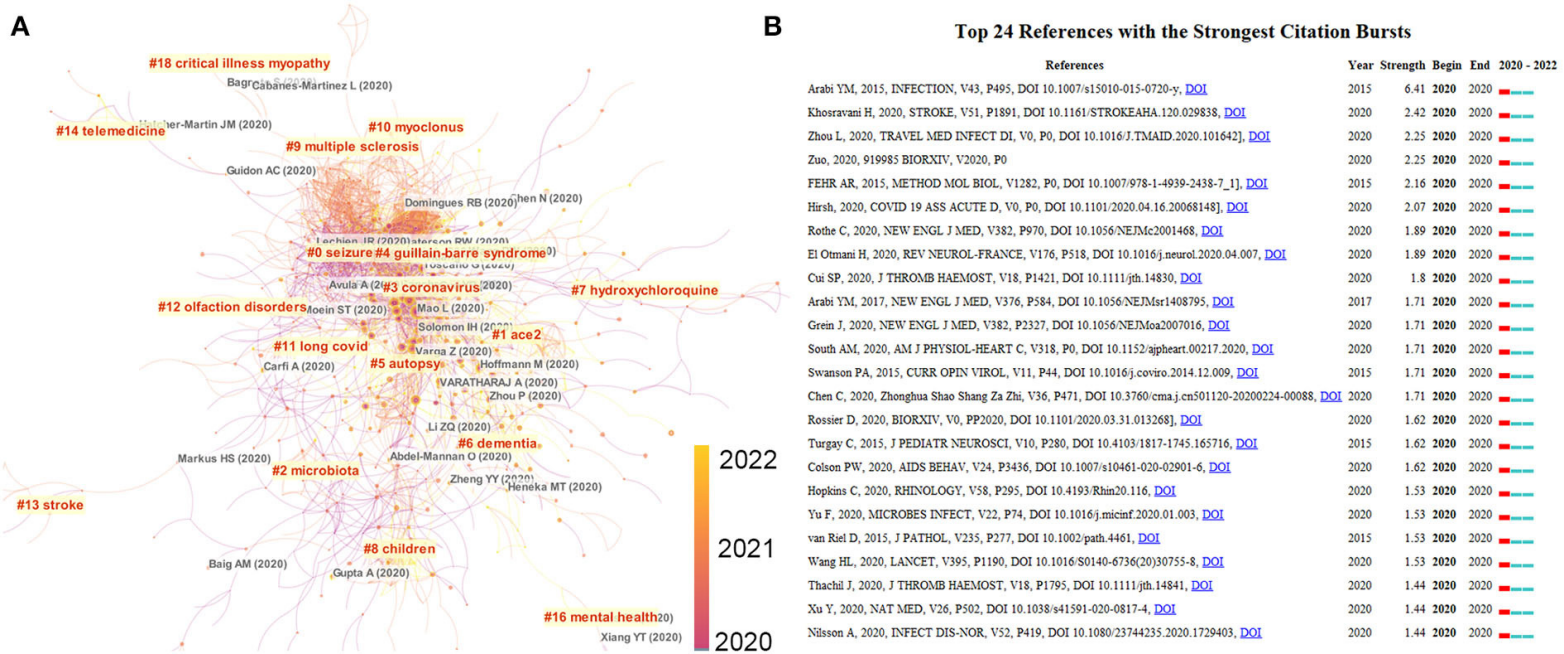
Analysis of references of neurology-related research on COVID-19. **(A)** Reference clusters named by CiteSpace (find cluster, keyword, LLR). **(B)** References citation burst visualized by CiteSpace. The top 24 references with the strongest citation bursts of neurology-related research on COVID-19 from 2019 to 2022. The red segment of the blue line denotes the burst duration of the reference.

**Figure 7:**
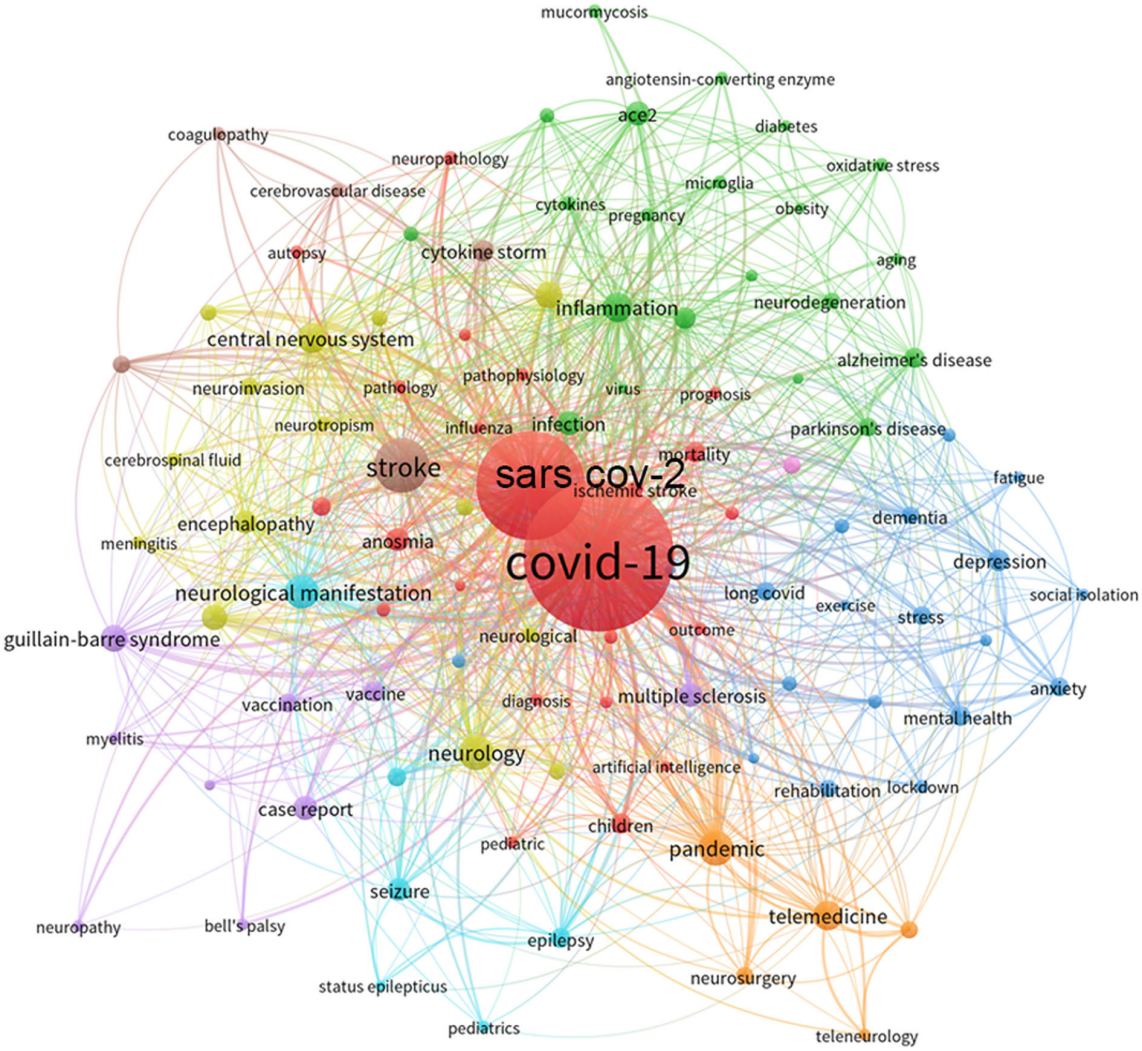
Co-occurrence networks of keywords visualized by VOSviewer. The keywords were clustered into six groups according to their color. Large nodes represent keywords with high frequency.

To present the key references and hot topics in the field, we conducted a reference analysis with CiteSpace and a keyword co-occurrence analysis with VOSviewer. As shown in Figure 6A, CiteSpace identified key references in neurology-related research on COVID-19 for different time periods. The main topic focused on “ethics, autopsy, and telemedicine” in 2020 and shifted to the various COVID-19-related neurological complications in 2021, such as stroke, Guillain–Barre syndrome (GBS), epilepsy, and long COVID. The most cited references are considered to be the basis for the frontier direction in a given field. Therefore, the reference analysis in CiteSpace was used to identify key references related for neurology-related research on COVID-19. In Figure 6B, the top 24 references with the highest citation burst were identified. The highest citation burst reference was generated by Arabi et al. The authors described three patients infected with Middle East respiratory syndrome coronavirus (MERS-CoV)-associated neurological syndrome in 2015. It is emphasized that the CNS may also be a target of MERS-COV (26). The second highest citation burst was produced by Khosravani et al. In this article, the authors introduced a framework for the management of hyperacute stroke during the COVID-19 pandemic, providing guidelines for researchers and neurologists, neurosurgeons, and policymakers to manage patients with COVID-19 and stroke (27). In VOSviewer, we performed a keyword occurrence analysis (Figure 7). The minimum number of occurrences was 20. Thesaurus (Supplementary Table S1) was used to remove duplicate keywords, such as COVID 19. Novel coronavirus 2019 were replaced by COVID-19. Finally, 104 keywords were selected from a total of 9,443 keywords. The top six most frequent keywords were “COVID-19 (*n* = 3,367), SARS-CoV-2 (*n* = 1,780), stroke (*n* = 244), neurology (*n* = 181), neurological manifestation (*n* = 168), and telemedicine (*n* = 137)”. As shown in Figure 7, seven research directions were formed based on these keywords: (1) cerebrovascular diseases associated with COVID-19 (brown): stroke, ischemic stroke, cytokine storm, and coagulation disorders; (2) neurodegenerative diseases associated with COVID-19 (green): inflammation, ACE2, Alzheimer's disease, Parkinson's disease, and oxidative stress; (3) COVID-19-related encephalitis and encephalopathy (yellow): central nervous system, brain, encephalitis, encephalopathy, and neuroinvasive; (4) neuroimmune complications associated with SARS-CoV-2 and its vaccine (purple): Guillain–Barre syndrome, multiple sclerosis, Bell's palsy, vaccine, and case report; (5) long COVID and mental problems associated with COVID-19 (dark blue): depression, mental health, stress, anxiety, long COVID, dementia, and rehabilitation; (6) neurological manifestations in children associated with COVID-19 (light blue): seizures, epilepsy, pediatrics, and persistent epilepsy; and (7) telemedicine during the COVID-19 pandemic (orange): pandemic, telemedicine, telemedicine, and tele-neurology.

## Discussion

Although the proportion of neurological disease caused by COVID-19 infection remains small, with a multitude of people infected, the overall number of neurological patients and their associated health burden may be large (3). The number of publications increased rapidly in 2021 compared to that in 2020, suggesting that researchers have been mobilized to uncover the neurological features of the COVID-19 pandemic. Its neurological manifestations, control measures, and acute and long-term complications have also attracted considerable attention worldwide. However, enthusiasm for neurology-related research on COVID-19 may decline, with only 687 publications in the first 4 months of 2022. With the COVID-19 pandemic lasting for 2 years, people seem to have passed the most panic phase, and researchers have lowered their interest in COVID-19. This can be confirmed by the new COVID-19 variant Omicron, which has a character of faster transmission, lower fusogenicity, and reduced pathogenicity (28).

### Analysis on countries and institutions

The existing publications include 142 countries/territories and 7,684 institutions, indicating that the neurology-related research on COVID-19 attracted the attention of global scholars. Among the top 20 prolific countries, 12 are from developed countries and eight are from developing countries. Of the top 10 prolific institutions, eight are from developed countries and only two are from developing countries. The United States, as the most productive country, dominates the field, with 1,677 publications (31.4% of the total) and 31,371 total citations (44.8% of the total). Previous studies have shown that the number of publications by country is positively correlated with the duration of the COVID-19 pandemic (29). The United States is one of the countries hardest hit by the COVID-19 pandemic. As of 24 April 2022, more than 80 million confirmed cases and more than 986,000 deaths have been reported in the United States. More importantly, inter-institution collaboration is more frequent in the United States (Figures 4A,B), which corroborates its dominate role in this field. Italy ranks second in terms of the number of publications. Since the emergence of the pandemic in China, there has been an outbreak of COVID-19 in Europe, particularly in Italy and the United Kingdom. This could explain why a large number of articles have been published in Italy as well. The University of Oxford, University College London, the UCL Queen Square Institute of Neurology, and King's College London collaborate closely. This may partly explain why the United Kingdom ranks third, with 530 articles, but second in terms of total citations. Other bibliometric analyses on COVID-19 (e.g., urology (12), rheumatology (13), and pediatrics (30)) also reported the leading position of the United States, Italy, and the United Kingdom, which is consistent with our results. In terms of international collaborations, the United States ranks first in terms of the number of collaborations with different countries, especially with Canada. By contrast, six of the top 10 highest collaborative countries are located in Europe, suggesting that different countries prefer to cooperate with those countries that are geographically close, especially during social isolation policies.

Among the top 10 prolific institutions, four are located in the United States, suggesting the United States conducted more research on neurology-related work on COVID-19 and the U.S. institutions have relatively tighter cooperation in this field (Figures 4A,B). The Huazhong University of Science and Technology (China) ranked first in terms of the total citations. Mao et al. (Tongji Medical College, Huazhong University of Science and Technology, China) first reported that 36.4% of hospitalized patients with COVID-19 had neurological manifestations. This article was also the most cited article in this field, accounting for 80% of the total citations of the Huazhong University of Science and Technology. COVID-19 was first reported in Wuhan, China. There is no doubt that the city's experience has provided researchers with valuable information regarding the characterization and early treatment of COVID-19-related neurological disorders (31, 32).

### Analysis on authors

The most prolific authors (*n* = 14 publications) were Josef Finsterer from the University of São Paulo (Austria), David Garcia-Azorin from the University Clinical Hospital of Valladolid (Spain), Nima Rezaei from the Tehran University of Medical Sciences (Iran), and Henrik Zetterberg from the UCL Queen Square Neurological Institute (UK). In terms of citations and citations per article, Tom Solomon ranked first, with 1,100 total citations and 185 citations per article. Focusing on these scholars may help new researchers design their studies and grasp the hot spots in neurological on COVID-19. For example, Josef Finsterer focuses on COVID-19-related cranial neuropathy (33, 34) and COVID-19-related GBS (35–37). David Garcia-Azorin focuses on post-pandemic neurological syndromes (e.g., persistent headache (38, 39) and insomnia (40)). Nima Rezaei first mainly focused on neurological complications of COVID-19 (41, 42) and then shifted to the neurological side effects of COVID-19 vaccination (43). Henrik Zetterberg focused on exploring potential neurochemical biomarkers and underlying mechanisms of COVID-19-related neuropathy (44–46), Tom Solomon was the most cited author because he published two articles in *Lancet Neurology* (3) and *Lancet Psychiatry* (5) that give readers a comprehensive understanding of the neurological and neuropsychiatric complications of COVID-19.

### Analysis on journals

*Neurological Sciences* and *Frontiers in Neurology* were the top two prolific journals listed as Q2 by the Journal Citation Reports (JCR). In terms of impact, *Journal of Neurology* ranked first, with 1,535 total citations and 23.6 citations per article, and also ranked Q2 by the JCR. However, most of the highly cited articles were published in the top journals such as *JAMA Neurology* (4), *Lancet Neurology* (3), *Lancet Psychiatry* (47), and *Brain Behavior and Immunity* (48). None of the most cited articles appear in the list of the top 10 most prolific journals. This partly reflects the position of the leading journals in the field of COVID-19-related neurology. Therefore, researchers can read the classic articles in these top journals to build their knowledge base in the field and stay updated with these prolific journals.

### Analysis on keywords and research frontiers

There can be no scientific investigation without prior knowledge. Keyword analysis and reference analysis help researchers gain quick insights into a given field (49). In this study, VOSviewer was used for keyword co-occurrence analysis and CiteSpace for reference analysis. A total of 104 keywords appeared more than 20 times and were clustered into seven groups by VOSviewer. CiteSpace identified 13 groups, of these seven groups represented popular topics for neurology-related research on COVID-19.

#### Group 1 COVID-19-related cerebrovascular diseases

The primary keywords include stroke, ischemic stroke, cytokine storm, and coagulopathy. In addition to respiratory distress symptoms in COVID-19 patients, neurological manifestations are the most common presentations. Among them, hemorrhagic stroke and ischemic stroke (IS) (50, 51) are also the most common and severe neurological complications after SARS-CoV-2 infection. Clinical studies have reported that the IS incidence ranges from 0.1 to 6.9% among hospitalized patients with COVID-19 (52). The IS incidence is higher in COVID-19 patients than in non-COVID-19 patients, especially in those with severe infections and ICU admissions (53). Moreover, COVID-19 patients with IS are younger and have more severe neurological symptoms than non-COVID-19 patients (51). Apart from arterial cerebral disorders, cerebral venous thrombosis (CVT) has also been found in some cases (54, 55). Because of mass vaccination, vaccine-induced cerebral venous sinus thrombosis (CVT) has also reported by several researchers. D'Agostino et al. reported a rare case of CVT and disseminated intravascular coagulation 12 days after COVID-19 vaccination (56). Schultz et al. (57) found that a 0.55% incidence of CVT within 1 month since the first dose vaccination and that women are more likely to develop CVT after vaccination. Mechanistically, RAS system imbalance, endothelial damage, cytokine storm, hypercoagulable state, and impaired immune function are suspected to contribute to acute cerebrovascular events and deserve more research in future (32).

#### Group 2 COVID-19-related neurodegenerative diseases

The main keywords include inflammation, ACE2, Alzheimer's disease (AD), Parkinson's disease (PD), and oxidative stress. Although there is insufficient evidence that PD and AD *per se* increase the risk of COVID-19 (58), COVID-19-related anosmia is a common feature of early PD. Blood leakage of the blood brain barrier in AD is also a target for COVID invasion. In addition, coronaviruses can be detected in the CNS of patients with PD and AD (59). All of these shows the close relationship between COVID-19 and AD or PD. Mechanically, cytokine storms and excessive oxidative stress induced by COVID-19 may trigger deleterious effects of immune responses, accelerate or exacerbate pre-existing cognitive deficits, or induce neurodegenerative diseases. Therefore, Verkhratsky et al. hypothesized that a population might be at risk of developing degenerative diseases after COVID-19 (60). Studies have shown that symptoms of movement disorders and dementia have been identified as risk factors for mortality in COVID-19 patients compared to AD and PD themselves (61). Quarantine may lead to a lack of motivation, physical disability, and increased stress and anxiety in patients with PD or AD, preventing them from engaging in active lifestyles. In addition, patients with PD and AD require routine clinical visits for physical assessment and medication modification. However, the lockdown and social isolation makes it hard during this particular time. As a result, numerous studies have explored telemedicine, transforming face-to-face clinical consultations into virtual physical examinations and consultations for patients with AD and PD (62, 63). For this, the International Parkinson's and Movement Disorders Association developed a practical step-by-step guide for implementing telemedicine in movement disorders clinics on its website (https://www.movementdisorders.org/MDS/About/Committees-other-groups/telemedicine-in-your-movement-obstacle-practice-A-Step-by-Step-Guide.htm).

#### Group 3 COVID-19-related encephalitis and encephalopathy

The main keywords include central nervous system, brain, encephalitis, encephalopathy, and neuroinvasive. Moriguchi et al. described the first case of meningitis and encephalitis associated with COVID-19, which occurred in a 24-year-old Japanese man. Interestingly, COVID-19-specific RNA was not detected in a nasopharyngeal swab but in the patient's cerebrospinal fluid (CSF) sample (19). By contrast, Kremer et al. reported a French multicenter cohort study involving 64 patients with confirmed neurological manifestations of COVID-19. The incidence of encephalitis was 13%, and no positive COVID-19-specific RNA was found in the CSF of these patients (64). Regarding encephalopathy, Meppiel et al. reported COVID-19-related encephalopathy in 30.2% (67/222) of patients. The authors hypothesized that toxic and metabolic causes, immune dysfunction, antiviral drugs, and hypoxia, rather than the virus itself, may cause COVID-19-related encephalopathy (65). Rutkai et al. infected SARS-CoV-2 on eight non-human primates *via* aerosol or multiple routes of exposure (e.g., conjunctival, nasal, pharyngeal, and intratracheal routes). The authors found that neuroinflammation, microhemorrhages, cerebral hypoxia, hypoxic-ischemic injury, neuronal degeneration, and apoptosis in the brains of these primates. They demonstrated the neuroinvasive nature of SARS-CoV-2, and these pathological findings may help provide insights into the neurological symptoms associated with long COVID (2).

#### Group 4 SARS-CoV-2 and its vaccine-related neuroimmune complications

The main keywords include Guillain–Barre syndrome (GBS), multiple sclerosis (MS), Bell's palsy, vaccine, and case report. The main neuroimmune disorders related to COVID-19 were GBS, MS, and Bell's palsy, all of which were characterized by demyelination and inflammation (66). Ottaviani et al. reported a woman who suffered from rapidly progressive flaccid paralysis and unilateral facial neuropathy after infected with SARS-CoV-2. Coronavirus was detected in a nasopharyngeal swab but was negative in her CSF (67). In agreement with this, in the first 6 months after the COVID-19 outbreak, Uncini et al. (68) reviewed 42 patients with COVID-19-related GBS, all of which were diagnosed with albuminocytological dissociation, rather than PCR positivity for COVID-19-specific RNA in CSF. It appears that the GBS occurrence after SARS-CoV-2 infection is not due to direct viral invasion of the nerve or CNS but rather due to immune dysfunction after COVID-19 infection. Future studies should compare patients with COVID-19-associated GBS to those with non-COVID-19-associated GBS over the same period and determine whether the incidence of GBS is elevated in COVID-19 patients in large cohorts. In the case of MS, COVID-19 does not appear to stimulate the initiation of MS. However, many researchers were interested in identifying whether the immunomodulatory treatment for COVID-19 has a negative effect on MS patients with COVID-19 infection. Loonstra et al. reported a Dutch multi-cohort study involving 86 Dutch MS patients. They found no significant negative effects of immunosuppression on patients with MS (69). Kovvuru et al. (37) also reported the same results. Immunosuppressive therapy does not make MS patients more susceptible to COVID-19. In addition to the most occurred vaccine-induced immunothrombotic events, Bell's palsy, encephalomyelitis, GBS, and transverse myelitis have been reported after COVID-19 vaccination (70). However, the authors noted that these immune-mediated neurological outcomes are rare in the vaccinated people when compared to the observed risks associated with unvaccinated COVID-19 people. Most of neurological complications related to SARS-CoV-2 vaccination are case reports. COVID-19-related mortality can currently be reduced most effectively through vaccination (71). Neurologists and policymakers should minimize reporting adverse effects related to COVID-19 vaccination and build public trust in vaccination programs to improve vaccination rates and curb the spread of infection.

#### Group 5 Long COVID and mental health problems

The main keywords include depression, mental health, stress, anxiety, long covid, dementia, and rehabilitation. After the initial surge of infection, concerns about acute mortality and complications of COVID-19 shifted to managing the long-term disease sequelae in survivors. As a result, post-acute COVID-19 syndrome (also known as long COVID) becomes a common syndrome. Davis et al. (72) reported the results of symptoms after 7 months of infection with COVID-19 in an international research containing 56 countries and 3,762 patients. The most common symptoms include fatigue (86.7%), post-exercise discomfort (85.9%), and cognitive dysfunction or memory issues (88%). These patients continue to suffer from the burden of these symptoms in their daily lives and are unable to return to the same level of work as they did before SARS-CoV-2 infection even months after infection. Interestingly, Duarte Romero et al. (73) reported the “long COVID” outcomes of 969 patients with severe COVID-19 after 6 months. Only 33% patients develop neurological and mental health problems. In addition, the authors found that women had a higher frequency of headaches and mental health problems. Lombardo et al. (74) reported the outcomes 1 year after COVID-19 infection, with the most prevalent symptoms being fatigue (52%), pain (48%), and sleep disturbance (47%). In addition, Kim et al. (75) reported the results of a 1-year follow-up research containing 241 patients with SARS-CoV-2 infection in Korea. The main symptoms are inattention, cognitive dysfunction, forgetfulness, depression, fatigue, and anxiety. In addition, 5.0% of the patients were receiving outpatient treatment for such symptoms after the infection was over. Older age, female gender, and disease severity were identified as risk factors for persistent neuropsychiatric symptoms. Petersen et al. (76) investigated the 8-month follow-up results of long COVID in 226 non-hospital individuals with confirmed COVID-19 diagnosis. The most common symptoms were fatigue (16%), and smell (17%), and taste (14%) dysfunction. Long COVID was more common in people taking medications every day. As for the mechanism, Dani et al. (77) proposed that long COVID may be associated with viral or immune-mediated disruption of the autonomic nervous system, leading to orthostatic intolerance syndrome. Newell et al. (78) concluded that immunological misfiring, inflammatory storms, and persistent inflammation played a key role in long COVID. However, there is a lack of concrete and consistently definition and characterization of “long COVID”. The reported prevalence of the so-called “long COVID” varies greatly. However, all physicians should be able to recognize this condition and comprehend its symptom burden. Moreover, with an increasing number of COVID-19 patients developing post-COVID manifestations, there is an urgent need to define the formal clinical “long COVID” and identify affected individuals early enough to provide the most appropriate and effective treatment.

#### Group 6 COVID-19-related neurological manifestations in children

The main keywords include seizures, epilepsy, pediatrics, and persistent epilepsy. Children infected with COVID-19 usually remain asymptomatic after infection, but some infected children will develop life-threatening multisystem inflammatory syndromes (MIS-C) (79). In addition to MIS-C, children with COVID-19 often develop neurological manifestations. A multicenter study of children diagnosed with MIS-C and COVID-19 showed that 5% of the children suffered severe neurological complications (e.g., seizures, coma, encephalitis, demyelinating disease, and aseptic meningitis). Among these neurological conditions, seizures were the most common reason for children visiting hospital (80). Kurd et al. (81) pointed out that seizures occurred in an early phase once children were infected with SARS-CoV-2 and may be the main manifestation of acute COVID-19 in children (11/175). Similarly, Dilber et al. (82) reported results of Turkey, where seizures (18/382) were the most common cause of hospitalization in children with COVID-19. In addition to the incidence of seizures attracted the interest from neurologists, the management of seizures also poses a significant challenge to neurologists and caregivers. Davico et al. (83) noted that during the COVID-19 pandemic, the hospital emergency visits due to childhood seizures decreased by 37% compared to those in the previous period. Furthermore, because of social isolation and lockdown policy, a large proportion of children with epilepsy have difficulties with regular clinic visits and medication refills (84). Panda et al. described their experience in providing medical advice to children with epilepsy *via* telephonic consultation during the pandemic. The results showed that 96% of caregivers were satisfied with the quality of this telemedicine service (85). Another study from India also found that video tele-consultation using cell phones improved the management of patients with epilepsy (86). Perhaps, telemedicine may be actively explored as an alternative strategy for the management and supervision of treatment of epilepsy patients in future.

#### Group 7 Telemedicine during the COVID-19 pandemic

The main keywords include epidemic, telemedicine, telehealth, and tele-neurology. Telemedicine refers to the delivery of medical care to a patient by a physician or other healthcare workers from a distance (87). The need for “social distance” and “lockdown policy” during the COVID-19 pandemic created a surging demand in the number of tele-neurology visits (88). Grossman et al. (89) described the transition of their neurology department from a face-to-face clinic to a virtual neurology practice in response to the COVID-19 pandemic. In addition, they performed a virtual neurological examination using the electronic medical record applications. Their experience suggests that changes in government rules, regulations, and payer-driven reimbursement policies during the COVID-19 period contributed to telemedicine. However, Fasano et al. (90) and Mok et al. (91) pointed out that telemedicine is only available in a few cases and limited to email or telephone contact. The authors advocate for remote management of patients with chronic neurological conditions, especially those with advanced dementia patients (e.g., AD and PD). Rametta et al. (92) analyzed 2,589 child tele-neurology visits during the COVID-19 pandemic. The results showed that 93% of clinicians (1,200/1,286) were satisfied with telemedicine services, and 89% (1,144/1,286) recommended telemedicine as an integral part of follow-up care after the COVID-19 pandemic. However, in a survey of telepractice visits to pediatric epilepsy clinics, physicians (79.9%) were less satisfied with telepractice than parents (91.3%) who used the telephone for their children remote encounters (93). Challenges to the widespread use of telemedicine in neurology clinics include (1) the lack of effective clinical examinations; (2) increased social isolation; (3) difficulties with patients with cognitive impairment; (4) workflow and technology availability challenges; and (5) legal issues and reimbursement problems (94). As the COVID-19 pandemic has caused worldwide social dislocation and operational and economic dysfunction, and its impacts on routine healthcare delivery may last for a long time, healthcare planners, policymakers, and academics should address these problems in future.

##### Limitations

First, we only extracted data from the WoSCC to meet the data formatting standards of visualization tools such as CiteSpace and VOSviewer. However, the WoSCC database, as one of the most widely available and recognized global resources, has been used in several previous high-quality bibliometric studies (95, 96). Second, this study produced a language and article-type bias as we only selected “English” literature published as “original articles or reviews”. Although “English” remains the most common language for academic publications worldwide, several articles have been published in non-English languages, such as Chinese, Japanese, and Portuguese. We excluded documents such as letters to the editor and editorials because the detailed information (e.g., authors, affiliations, keywords, and cited references) is often incomplete in these publication types. Third, total citations and citations per article are affected by time and remain controversial as a comprehensive indicator of the quality of an article or an author. Similarly, the number of publications is not the only indicator of a journal's impact or activity as other metrics (e.g., impact factor, SNIP, CiteSpace, and SJR) are widely used. Fourth, articles published in 2022 only cover the first 4 months of the year, and database updates may result in discrepancies. However, we believe that the low citation frequency of new publications has less impact on our conclusions.

## Conclusion

This study provides researchers with valuable information on publication trends, potential collaborations, topical issues, and frontier research topics in neurology-related research on COVID-19. During the COVID-19 pandemic, there has been a growing interest in this field. Research topics have shifted from “ethics, autopsy, and telemedicine” in 2020 to various neurological conditions related to COVID-19 in 2021, such as stroke, AD, PD, GBS, MS, childhood seizures, and long COVID. The following three topics deserve further research in future: tele-neurology during the COVID-19 pandemic, COVID-19-related neurological complications and mechanisms, and long COVID.

## Data Availability Statement

The original contributions presented in the study are included in the article/Supplementary material, further inquiries can be directed to the corresponding author/s.

## Author contributions

QZ conceived of the study, participated in its design, and drafted the manuscript. JL was involved in the study design, obtained data and contributed to interpretation, and helped to draft the manuscript. LW provided the theoretical frameworks and performed much of the editing of the manuscript. All authors read and approved the final manuscript.

## Funding

This research was supported by National Key R&D Program of China (No. 2020YFC2005401), National Natural Science Foundation of China (No. 81902551), and Natural Science Foundation of Hunan Province (No. 2021JJ31072).

## Conflict of interest

The authors declare that the research was conducted in the absence of any commercial or financial relationships that could be construed as a potential conflict of interest.

## Publisher's note

All claims expressed in this article are solely those of the authors and do not necessarily represent those of their affiliated organizations, or those of the publisher, the editors and the reviewers. Any product that may be evaluated in this article, or claim that may be made by its manufacturer, is not guaranteed or endorsed by the publisher.
