# Supplementary material for: A bibliometric analysis of COVID-19 publications in neurology by using the visual mapping method
Source: Front Public Health. 2022 Jul 26;10:937008. doi: 10.3389/fpubh.2022.937008 (PMC9362596; doi:10.3389/fpubh.2022.937008)
Supplement: Supplementary file 1 [file Table_1.pdf]

Table S1. Thesaurus

label	replace by
coronavirus disease 2019	covid-19
coronavirus disease 2019 (covid-19)	covid-19
covid	covid-19
covid 19	covid-19
covid-19 pandemic	covid-19
coronavirus infections	covid-19
covid-19 pandemic	covid-19
health care workers	health care worker
coronavirus	sars-cov-2
alzheimers-disease	alzheimer's disease
blood-brain-barrier	blood-brain barrier
central-nervous-system	central nervous system
cns	central nervous system
cerebrospinal-fluid	cerebrospinal fluid
converting enzyme 2	ace2
eeg	
guillain-barre-syndrome	guillain-barre syndrome
infections	infection
ischemic-stroke	ischemic stroke
multiple-sclerosis	multiple sclerosis
parkinsons-disease	parkinson's disease
risk-factors	risk factors
seizures	seizure
system	
sars	sars-cov-2
sars-cov-2 infection	sars-cov-2
sars-cov2	sars-cov-2
nervous system	central nervous system
neurologic manifestations	neurological manifestation
neurological disorders	neurological diseases
neurological symptoms	neurological manifestation
neurological manifestations	neurological manifestation
severe acute respiratory syndrome coronavirus	sars-cov-2
